# Pan-Cancer Analysis of B4GALNT1 as a Potential Prognostic and Immunological Biomarker

**DOI:** 10.1155/2022/4355890

**Published:** 2022-07-28

**Authors:** Hang Yi, Yiwen Lin, Yutong Li, Yeqi Guo, Ligong Yuan, Yousheng Mao

**Affiliations:** ^1^Department of Thoracic Surgery, National Cancer Center/National Clinical Research Center for Cancer/Cancer Hospital, Chinese Academy of Medical Sciences and Peking Union Medical College, Beijing 100021, China; ^2^Department of Medical Oncology, National Cancer Center/National Clinical Research Center for Cancer/Cancer Hospital, Chinese Academy of Medical Sciences and Peking Union Medical College, Beijing 100021, China; ^3^Peking University Health Science Center, Peking University, Beijing 100191, China

## Abstract

**Background:**

Gangliosides act as important roles in tumor progression. B4GALNT1 is a key enzyme in ganglioside biosynthesis. B4GALNT1 expression is linked to tumorigenesis and the prognosis of tumor patients. Nevertheless, the role of B4GALNT1 in pan-cancer remains unclear.

**Methods:**

Several databases, including TCGA, GEO, GTEx, NCI-60, and TIMER, were searched. Methods including correlation analysis, Cox regression analysis, and Kaplan-Meier analysis were used to explore the expression pattern, prognosis, tumor infiltration pattern, genetics and epigenetics, and drug sensitivity of B4GALNT1 in pan-cancer patients from the above datasets.

**Results:**

B4GALNT1 was found to be aberrantly expressed in multiple types of tumors. The survival status of tumor patients was significantly related to B4GALNT1 expression, but the correlations were tumor-specific. Moreover, the expression of B4GALNT1 was associated with ImmuneScore and StromalScore in 21 and 27 tumor types, respectively. Also, B4GALNT1 was significantly associated with TMB, MSI, MMR, and DNA methylation. Additionally, the sensitivity of 9 drugs was correlated with the expression of B4GALNT1.

**Conclusion:**

A correlation of B4GALNT1 expression with prognosis exists in multiple types of cancers. In addition, B4GALNT1 expression may play a role in TME and tumor immunity regulation. Further investigation of the biological mechanisms of its different roles in tumorigenesis and clinical application as a biomarker is still required.

## 1. Introduction

Tumorigenesis is a complex process with numerous genes modified and correlated with potential molecular mechanisms, in which ganglioside biosynthesis process plays a vital role [1]. Due to the foundation of publicly available bioinformatic databases such as TCGA and GEO, opportunities have been offered to perform pan-cancer analyses of the genes that are possibly related to tumorigenesis [2].

In the ganglioside biosynthesis process, Beta-1,4-N-Acetyl-Galactosaminyltransferase 1 (B4GALNT1) works as the GD2/GM2 synthase, which is a key enzyme that transfers an N-acetylgalactosamine (GalNAc) to GD3/GM3 and forms GD2/GM2 [3]. Gangliosides are acidic glycosphingolipids (GSL) that contain one or more sialic acid residues [4] and have been proven to act an important role in the tumorigenesis of various types of cancers, including neuroblastoma [5], melanoma, lung cancers [4], clear cell renal cell carcinoma [6], and breast cancers [7]. These gangliosides are important molecules as they have complex functions in tumor cells, and previous studies have reported their role in cell adhesions and in mediating cytotoxic signals [8]. Moreover, specific gangliosides including GD2 and GM2 have been found to induce angiogenesis and anchorage-independent growth in tumorigenesis [9, 10]. The above findings emphasize the importance of gangliosides in tumorigenesis, and B4GALNT1, the key enzyme in the ganglioside biosynthesis, undoubtedly plays a major role in tumorigenesis and malignant progression. However, there is still a lack of pan-cancer evidence based on big clinical data on the correlation of B4GALNT1 with various types of tumors. Therefore, this study, for the first time, searched as many publicly available databases as possible and conducted an analysis of the pan-cancer role of B4GALNT1 in tumorigenesis. The potential molecular mechanism of B4GALNT1 in pan-cancer gene expression, prognosis, genetic alteration, immune infiltration, relevant cellular pathway, and sensitivity of targeted drugs for it was investigated.

## 2. Methods

### 2.1. B4GALNT1 Expression Analysis across Multiple Tumors and Corresponding Normal Tissues

The information on B4GALNT1 expression of 31 normal tissues was gathered from Genotype-Tissue Expression (GTEx) portal, and the data of 21 tumor cell lines were downloaded through the Cancer Cell Line Encyclopedia (CCLE) database. Data from The Cancer Genome Atlas (TCGA), an open-access gene expression database, was combined with data from GTEx to evaluate the comparison between 33 cancer tissues and normal tissues.

TIMER2 was used to study the different expressions of B4GALNT1 between tumors and corresponding normal tissues for diverse tumors or specific tumor subtypes in the TCGA database.

For a few tumors that barely have or have limited normal tissues (e.g., TCGA-LAML (acute myeloid leukemia)), the GEPIA2 (gene expression profiling interactive analysis, version 2) web-server was used to reveal the difference in expression between these tumor tissues and their corresponding normal tissues of the GTEx database.

Furthermore, information on B4GALNT1 expression among pan-cancers in different stages according to their pathological behaviors in the TCGA database was also obtained from GEPIA2, and the log2 (TPM (transcripts per million) + 1) transformed expression data was revealed in violin plots.

### 2.2. Survival Status and Prognosis Analysis

We obtained data from the TCGA database to reveal the correlation between B4GALNT1 expression and patient prognosis, including disease-free interval (DFI), disease-specific survival (DSS), overall survival (OS), and progression-free interval (PFI). We analyzed the survival in all 33 types of tumors, and the results were revealed through forest maps of univariate Cox regression analysis and Kaplan-Meier curves.

### 2.3. B4GALNT1 Genetic Alteration Analysis

The cBioPortal web was selected for the genetic alteration analysis of B4GALNT1. From this website, we obtained information of B4GALNT1 alteration frequency, mutation types, copy number alterations, and mutation sites in pan-cancers from the TCGA database. Moreover, data on prognosis for all TCGA cancers with and without B4GALNT1 genetic alterations were extracted from cBioPortal as well, which contained disease-free survival, overall survival, disease-specific survival, and progression-free interval differences.

### 2.4. Correlation of B4GALNT1 Expression with Immune Infiltration Characteristics

The TIMER2 web-server was used to assess the association between B4GALNT1 expression and immune infiltrations in pan-cancers of the TCGA database. The immune cells and tumor-related fibroblasts were chosen. The seven algorithms including TIMER, CIBERSORT, CIBERSORT-ABS, QUANTISEQ, XCELL, MCPCOUNTER, and EPIC were used for immune infiltration evaluations. A heat map and scatter plots were shown in the supplement to show the relationship of B4GALNT1 expression with immune cells and cancer-associated fibroblasts.

Immune and stromal scores were calculated using the ESTIMATE (Estimation and Stromal and Immune cells in Malignant Tumor tissues with Expression data) algorithm. Spearman's correlation method was then applied to analyze the association of B4GALNT1 expression with the above two scores. The Spearman's correlation analysis was also applied to assess the correlation of B4GALNT1 expression with the expression levels of 47 immune checkpoint genes.

### 2.5. Correlation between B4GALNT1 Expression and Tumor Mutational Burden, Microsatellite Instability, Mismatch Repair, and Immune Checkpoints across Cancers

“Maftools” R package was implemented to analyze the somatic mutation among the human pan-cancer data obtained from the TCGA database. We determined tumor mutation burden (TMB) as the total count of somatic mutations detected in the tumor. The MSI score, information of the 5 MMR genes, and methylation expression information were also obtained from the TCGA database. Spearman's correlation method was applied to evaluate the relationship of all the above information with the B4GALNT1 expression.

### 2.6. B4GALNT1-Related Gene Enrichment Analysis

The STRING tool was utilized to obtain the top 50 B4GALNT1-binding proteins. GEPIA2 was used to retrieve the top 100 B4GALNT1-related targeting genes based on the datasets of all TCGA tumors and normal tissues. The log2 TPM was used for the dot plot. In addition, the TIMER2 web server was used to apply the heat map data of the chosen genes.

Furthermore, we integrated the two sets of genes to conduct GO and KEGG analysis via the “clusterProfiler” R package. The results of the GO analysis contained biological process (BP), molecular function (MF), and cellular component (CC).

### 2.7. B4GALNT1-Related Sensitive Drug Analysis

The National Cancer Institute- (NCI-) 60 database was used to establish the drug sensitivity analysis of B4GALNT1. RNA-seq and compound activity data were achieved from the cellMiner database. NCI-60 is a publicly available database based on nine cancer types and 60 cancer cell lines, which includes mRNA expression level and their corresponding *z* scores of cell sensitivity data (GI50) after drug treatment. The Pearson correlation between each gene expression and the GI50 was calculated to explore the association of these genes with multiple drug sensitivity. FDA-approved drugs or drugs that were currently in clinical trials were selected in this drug sensitivity analysis. The R language software was also used in this analysis with the “Impute” package, “limma” package, “ggplot2” package, and “ggpubr” package.

## 3. Results

### 3.1. Gene Expression Analysis

**Figure 1:**
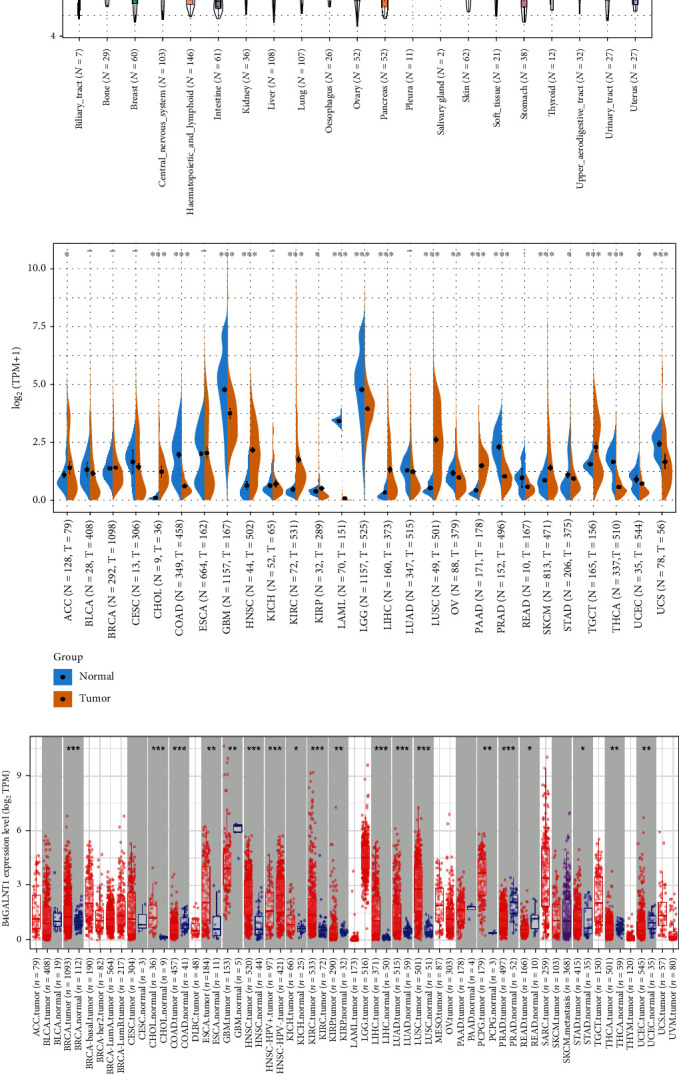
The expression levels of B4GALNT1 across cancers. (a) Data of GTEx database showed the expression levels of B4GALNT1 in normal tissues. (b) Data of CCLE database showed the expression levels of B4GALNT1 across different cancer lines. (c) Data of TCGA and GTEx database showed the expression of B4GALNT1 in diverse tumors and normal tissues. (d) Data of TCGA database using TIMER2 approach showed the expression levels of B4GALNT1 across multiple tumors and normal tissues. (e) Data of GTEx comparing tumor tissues and normal tissues in LAML LGG and UCS. (f) Data of GEPIA2 showed the expression levels of B4GALNT1 in different pathological stages of tumors.

The GTEx database was applied to evaluate the level of B4GALNT1 in 31 normal tissues. We found that the expression level of B4GALNT1 was relatively high in the brain and nerve tissues, which is consistent with the fact that B4GALNT1, as a key enzyme in ganglioside biosynthesis, takes an important role in the nervous system in previous studies [3]. The expression level was relatively low in the blood, kidney, liver, and pancreas tissues (Figure 1(a)). Moreover, we examined the expression level of B4GALNT1 in 21 tumor cell lines based on the data of the CCLE database. The expression of B4GALNT1 was different in each different cell line, but none was relatively high or low (Figure 1(b)). Furthermore, we combined the data from TCGA and GTEx databases to assess the differences of B4GALNT1 expression levels between 27 cancer types and corresponding normal tissues. Compared with normal tissues, B4GALNT1 was expressed higher in CHOL, HNSC, KIRC, LIHC, LUSC, PAAD, SKCM, TGCT (*P* < 0.001), ACC, and KIRP (*P* < 0.05), while the expression was relatively low in COAD, GBM, LAML, LGG, PRAD, THCA, UCS (*P* < 0.001), OV (*P* < 0.01), STAD, and UCEC (*P* < 0.05) than those in normal tissues (Figure 1(c)).

The TIMER2 approach was applied to analyze the expression status of B4GALNT1 among multiple types of cancers from the TCGA database. As shown in Figure 1(d), the expression level of B4GALNT1 in the tumor tissues of BRCA, CHOL, HNSC, KIRC, LIHC, LUAD, LUSC (*P* < 0.001), ESCA, PCPG (*P* < 0.01), KICH, and STAD (*P* < 0.05) is higher than that in the corresponding normal tissues, while B4GALNT1 was expressed lower in COAD, PRAD (*P* < 0.001), GBM, KIRP, THCA, UCEC (*P* < 0.01), and READ (*P* < 0.05) compared with normal tissues. In HNSC without HPV infection, the expression of B4GALNT1 was higher than that in HNSC tissues with HPV positive (*P* < 0.001).

After counting in the normal tissue of the GTEx dataset as controls, the expression differences of B4GALNT1 between the normal tissues and tumor tissues of LAML, LGG, and UCS were examined (Figure 1(e) (*P* < 0.05)), and the expression level was found to be relatively high in normal tissues.

The “Pathological Stage Plot” module of GEPIA2 was used to study the correlation between B4GALNT1 expression and the pathological stages of cancers, including BLCA, COAD, ESCA, KICH, KIRP, TCGT, LUAD, LUSC, LIHC, and UCEC (Figure 1(f), all *P* < 0.05). The results in these cancers indicated that the expression of B4GALNT1 presented with significance in different tumor stages, which reveals the possible relevance of B4GALNT1 expression and tumor progression.

### 3.2. Survival Analysis

**Figure 2:**
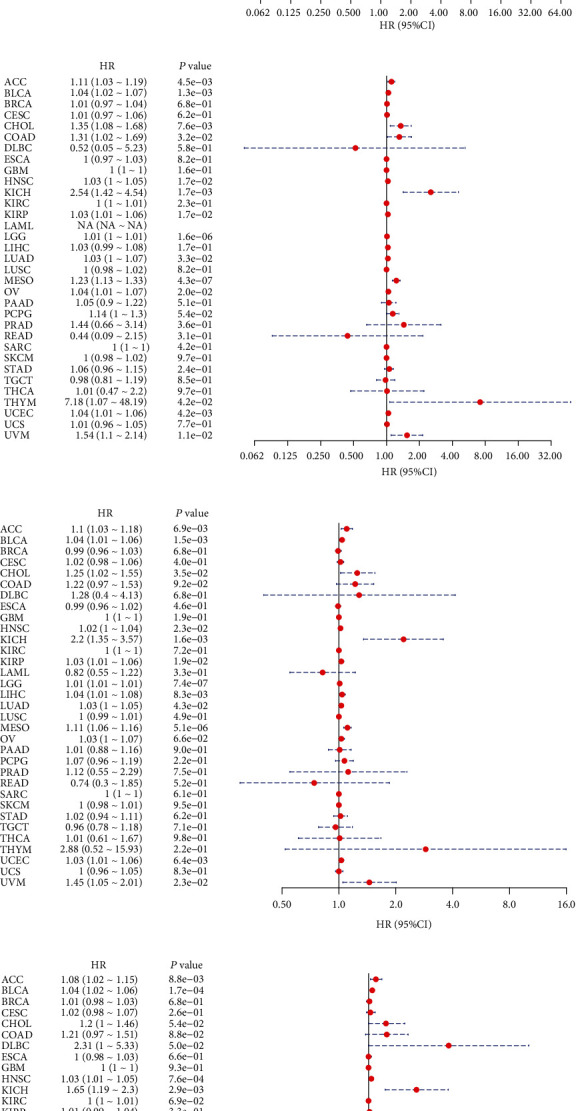
Univariate Cox analysis of (a) disease-free survival, (b) disease-specific survival, (c) overall survival, and (d) progression-free survival in association with B4GALNT1 expression in multiple tumors.

**Figure 3:**
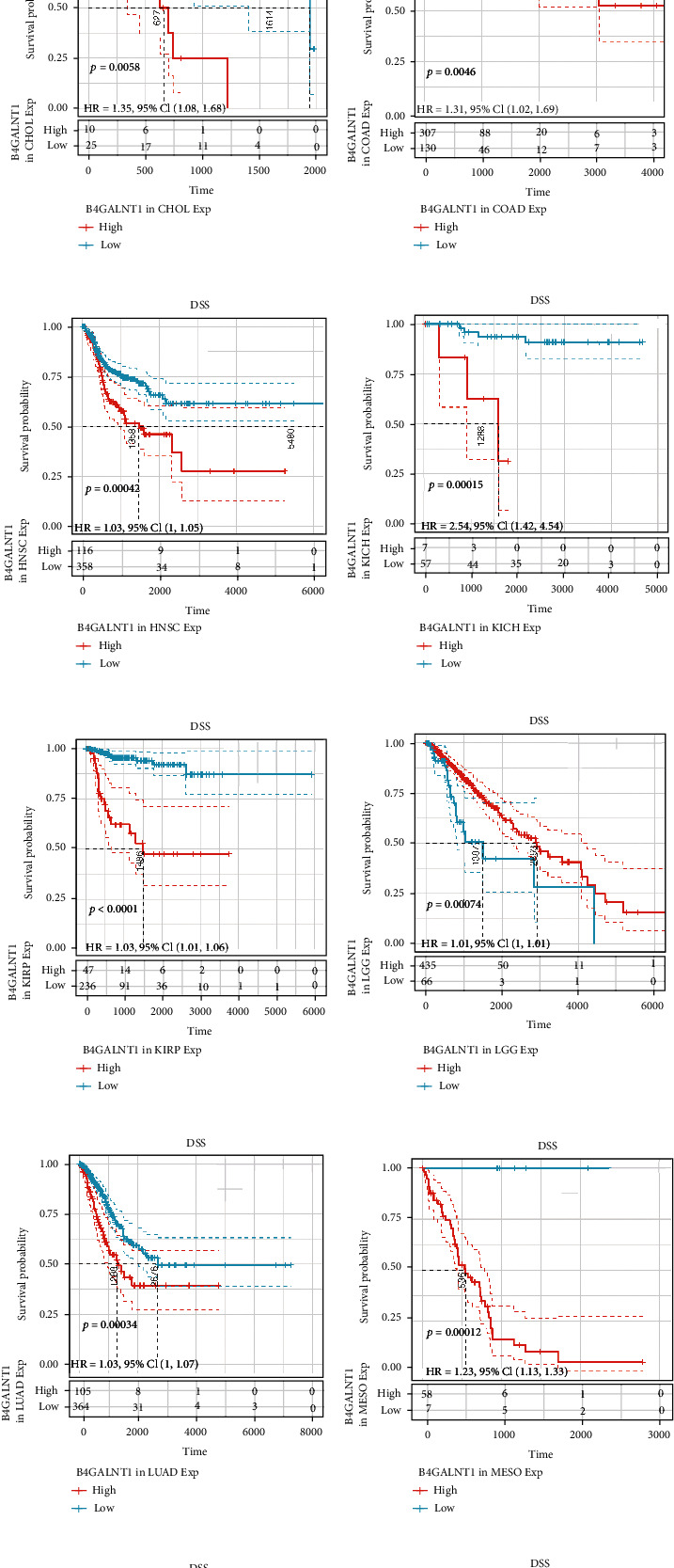
(a, b) Survival analysis of B4GALNT1 expression using Kaplan-Meier DFI curves in CHOL and THCA. (c–p)Survival analysis of B4GALNT1 expression using Kaplan-Meier DSS curves in ACC, BLCA, CHOL, COAD, HNSC, KICH, KIRP, LGG, LUAD, MESO, OV, THYM, UCEC, and UVM.

**Figure 4:**
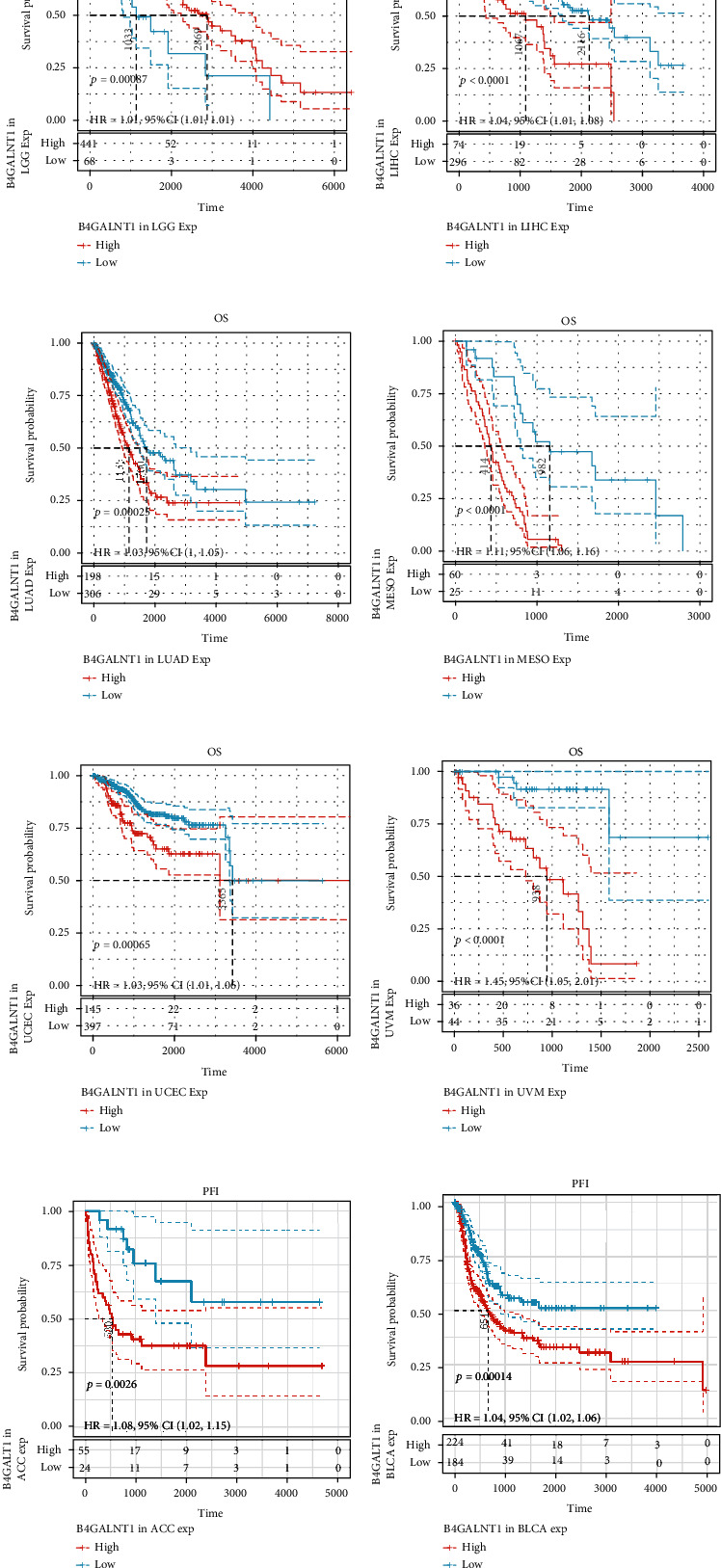
(a–l) Survival analysis of B4GALNT1 expression using Kaplan-Meier OS curves in ACC, BLCA, CHOL, HNSC, KICH, KIRP, LGG, LIHC, LUAD, MESO, UCEC, and UVM. (m–s) Survival analysis of B4GALNT1 expression using Kaplan-Meier PFI curves in ACC, BLCA, HNSC, KICH, LGG, MESO, and UVM.

B4GALNT1 expression presented with significant prognostic value across different types of tumors. We used Univariate Cox analysis to evaluate the association of B4GALNT1 with disease-free interval (DFI, Figure 2(a)), disease-specific survival (DSS, Figure 2(b)), overall survival (OS, Figure 2(c)), and progression-free interval (PFI, Figure 2(d)) among 33 types of cancers. The results in the forest maps indicated that B4GALNT1 affected DFI in CHOL (*P* = 0.039) and THCA (*P* = 0.048) (Figure 2(a)); the correlation of B4GALNT1 expression with DSS was also observed, and its higher expression led to poorer prognosis of multiple types of tumor, including ACC (*P* = 0.0045), BLCA (*P* = 0.0013), CHOL (*P* = 0.0076), COAD (*P* = 0.032), HNSC (*P* = 0.017), KICH (*P* = 0.0017), KIRP (*P* = 0.017), LGG (*P* = 1.6*e* − 06), LUAD (*P* = 0.033), MESO (*P* = 4.3*e* − 07), OV (*P* = 0.02), THYM (*P* = 0.042), UCEC (*P* = 0.0042), and UVM (*P* = 0.011) (Figure 2(b)); the expression of B4GALNT1 was related to the OS of patients in ACC (*P* = 0.0069), BLCA (*P* = 0.0015), CHOL (*P* = 0.035), HNSC (*P* = 0.023), KICH (*P* = 0.0016), KIRP (*P* = 0.019), LGG (*P* = 7.4*e* − 07), LIHC (*P* = 0.0083), LUAD (*P* = 0.043), MESO (*P* = 5.1*e* − 06), UCEC (*P* = 0.0064), and UVM (*P* = 0.023) (Figure 2(c)); the higher expression level of B4GALNT1 influenced the PFI of patients in tumors including ACC (*P* = 0.0088), BLCA (*P* = 1.7*e* − 04), HNSC (*P* = 7.6*e* − 04), KICH (*P* = 0.0029), LGG (*P* = 3.9*e* − 05), MESO (*P* = 3.8*e* − 04), and UVM (*P* = 0.0012). These results revealed that B4GALNT1 expression had impact on poorer prognosis of patients in the types of tumors mentioned above, especially in ACC, BLCA, CHOL, HNSC, KICH, LGG, MESO, and UVM. To further illustrate the prognostic potential of B4GALNT1, we divided the cancer cases into high-expression and low-expression groups according to the expression levels of B4GALNT1, mainly using the datasets of TCGA. Kaplan-Meier curves confirmed the above results (Figures 3(a)–3(p) and 4(a)–4(s)). The relationship between B4GALNT1 and the prognosis of patients across multiple types of tumors was concluded in Table 1. B4GALNT1 expression is related to poorer prognosis in patients, but the relationship is cancer-specific.

### 3.3. Genetic Alteration Analysis

**Figure 5:**
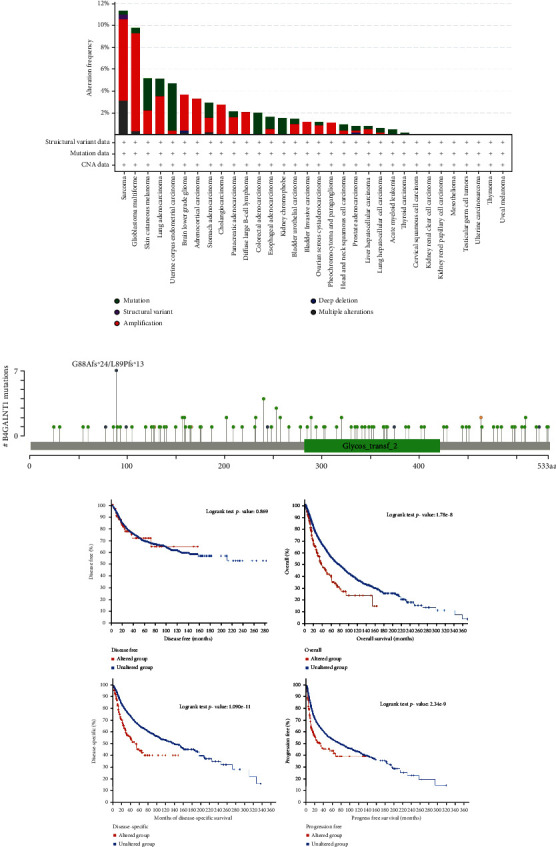
Genetic alteration analysis of B4GALNT1 in diverse tumor tissues. (a) The alteration frequency of different genetic alteration types of B4GALNT1 in diverse tumor samples. (b) Types and sites of B4GALNT1 genetic alteration. (c) Survival analysis of B4GALNT1 genetic alteration using Kaplan-Meier DFS, OS, DSS, and PFS in different types of cancers.

The genetic alteration status of B4GALNT1 in different tumor samples of the TCGA cohorts was evaluated. As shown in Figure 5(a), the highest alteration frequency of B4GALNT1 (>10%) appeared in patients with sarcoma, mainly presenting “amplification” as the primary type. The types and sites of the B4GALNT1 genetic alteration are further presented in Figure 5(b). Additionally, the potential association between genetic alteration of B4GALNT1 and the clinical survival status of cases with different types of cancers was explored. The data of Figure 5(c) indicated that all cases with altered B4GALNT1 showed better prognosis in overall (*P* = 1.78*e* − 08) and disease-specific (*P* = 1.09*e* − 11) and progression-free (*P* = 2.34*e* − 9) survival, but not disease-free (*P* = 0.869) survival, compared with cases without B4GALNT1 alteration.

### 3.4. Immune Infiltration Analysis and Immune Checkpoint Biomarkers

Tumor-infiltrating immune cells are strongly relevant with the initiation, progression, or metastasis of cancer as they are part of the most important components of the tumor microenvironment [11]. Previous studies have reported that immune cells can improve or obstruct therapeutic efficacy according to their different status within TME [12]. Meanwhile, tumor-related fibroblasts in the stroma of the tumor microenvironment were reported to take a significant role in regulating the function of various tumor-infiltrating immune cells. Therefore, we used the EPIC, MCPCOUNTER, XCELL, and TIDE algorithms to examine the potential association between the infiltration level of different types of immune cells and B4GALNT1 gene expression in various types of TCGA cancers (Supplement 1). We observed a statistically positive correlation between B4GALNT1 expression and the estimated infiltration value of cancer-associated fibroblasts for the TCGA tumors of BLCA-LumA, COAD, ESCA, HNSC, HNSC-HPV-, KIRC, PAAD, PRAD, READ, STAD, THCA, and THYM (Supplement 1A). The scatter plots showed the results of the above tumors in types of algorithms that presented with significant correlations (Supplement 1B**)**.

Tumor-infiltrating cells are considered independent predictors of sentinel lymph node status and patients' prognosis in cancers. Therefore, we studied the relationship between B4GALNT1 expression and the level of immune cell infiltration in 33 cancer types from the TIMER database. Results revealed that B4GALNT1 expression was significantly correlated with six types of infiltrating immune cells, including B cells, CD8 + T cells, CD4 + T cells, macrophage cells, neutrophil cells, and dendritic cells in BRCA, LGG, PAAD, PRAD, and THYM (Supplement 2).

**Figure 6:**
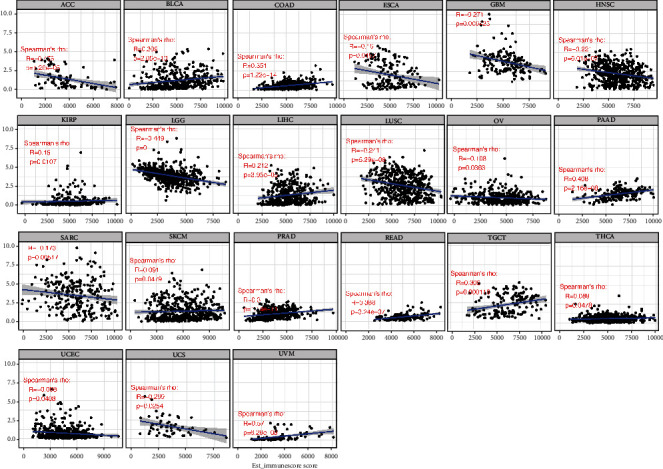
Correlation of B4GALNT1 expression with ImmuneScore in ACC, BLCA, COAD, ESCA, GBM, HNSC, KIRP, LGG, LIHC, LUSC, OV, PAAD, SARC, SKCM, PRAD, READ, TGCT, THCA, UCEC, UCS, and UVM.

**Figure 7:**
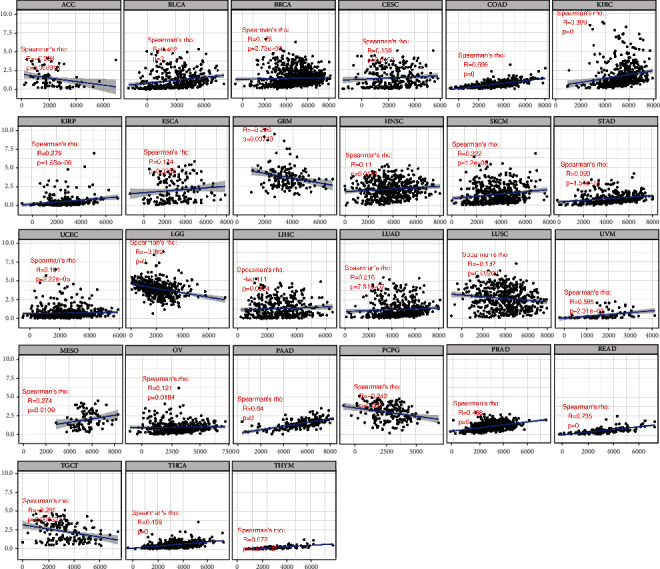
Correlation of B4GALNT1 expression with StromalScore in ACC, BLCA, BRCA, CESC, COAD, KIRC, KIRP, ESCA, GBM, HNSC, SKCM, STAD, UCEC, LGG, LIHC, LUAD, LUSC, UVM, MESO, OV, PAAD, PCPG, PRAD, READ, TGCT, THCA, and THYM.

We then combined ImmuneScore and StromalScore to further assess the link between B4GALNT1 and immune cell infiltration (Table 1). The results revealed that B4GALNT1 expression was positively correlated with ImmuneScore in BLCA, COAD, KIRP, LIHC, PAAD, SKCM, PRAD, READ, TGCT, THCA, and UVM, while it was negatively correlated in ACC, ESCA, GBM, HNSC, LGG, LUSC, OV, SARC, UCEC, and UCS (Figure 6). Meanwhile, B4GALNT1 expression showed a positive correlation with StromalScore in BLCA, BRCA, CESC, COAD, KIRC, KIRP, ESCA, HNSC, SKCM, STAD, UCEC, LIHC, LUAD, UVM, MESO, OV, PAAD, PRAD, READ, THCA, and THYM, but showed a negative correlation in ACC, GBM, LGG, LUSC, PCPG, and TGCT (Figure 7).

**Figure 8:**
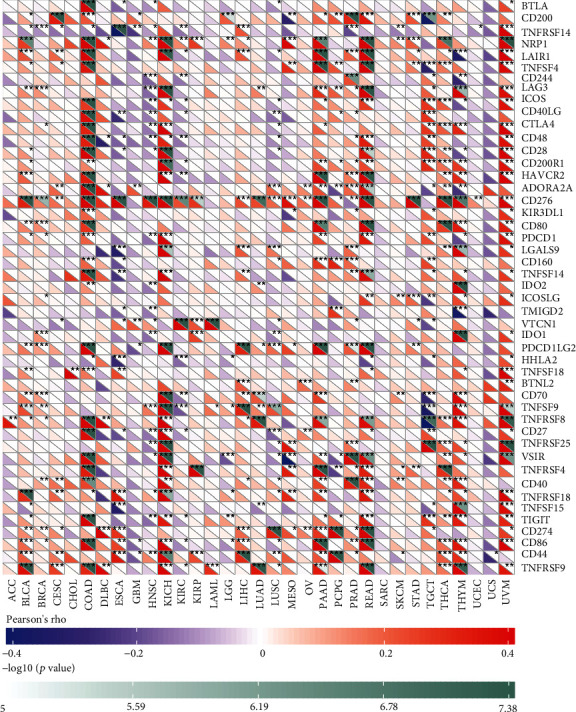
Correlation of B4GALNT1 expression with 47 common immune checkpoint gene expression across cancers. ∗*P* < 0.05, ∗∗*P* < 0.01, and ∗∗∗*P* < 0.001.

As the expression of B4GALNT1 showed a significant tumor-specific relationship with immune infiltration cell levels in multiple types of tumors, we further evaluated the relationship between B4GALNT1 expression and 47 common immune checkpoint genes (Figure 8). According to the results, B4GALNT1 expression showed correlations with 34 immune checkpoints genes in UVM, 33 in COAD, 29 in READ, 27 in KICH, and 26 in PAAD. The above results can lead to the conclusion that B4GALNT1 has great significance in tumor immunity regulation.

### 3.5. TMB, MSI, and MMR

**Figure 9:**
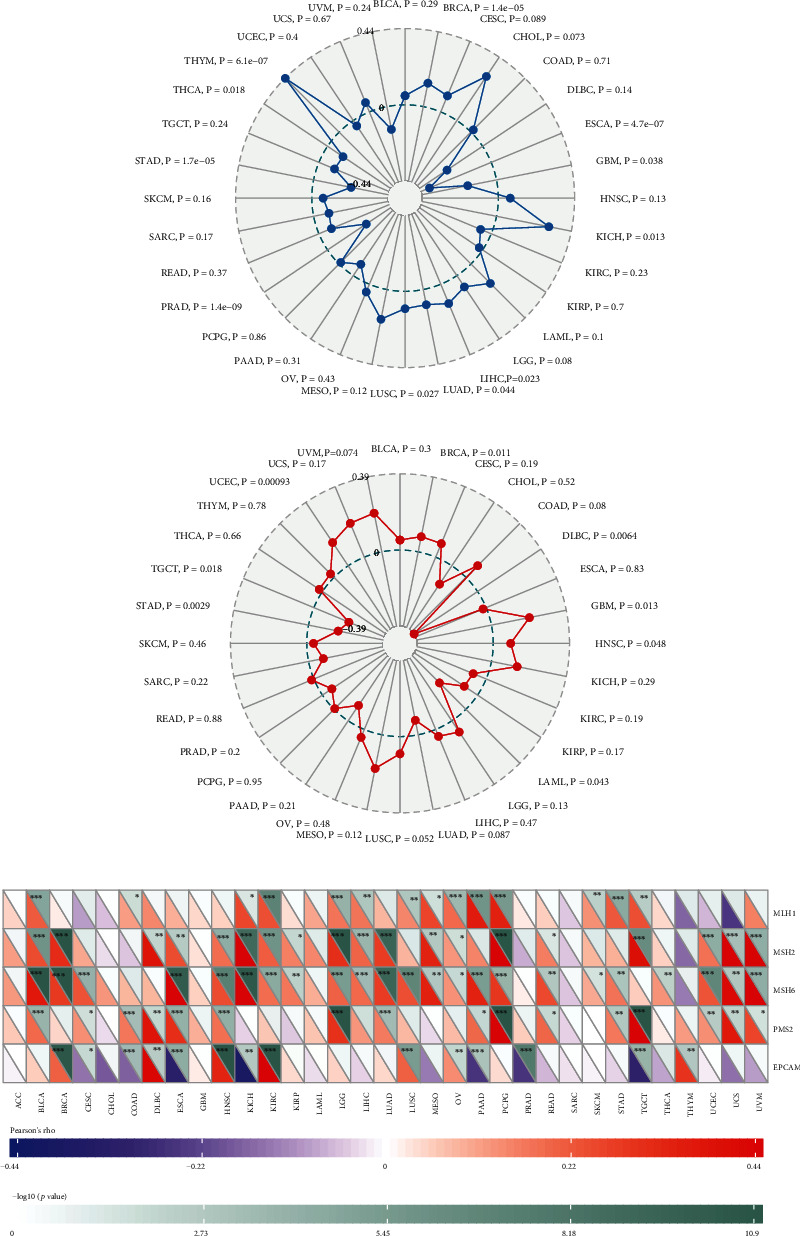
(a) The radar chart showed the correlation of B4GALNT1 expression with TMB across multiple cancers. (b) The radar chart showed the correlation of B4GALNT1 expression with MSI across multiple cancers. (c) The heat map showed the correlation of CTSL expression with the expression levels of five MMR genes across cancers. ∗*P* < 0.05, ∗∗*P* < 0.01, and ∗∗∗*P* < 0.001.

TMB represents the number of mutations per megabase of DNA that was sequenced in a specific type of tumor and has been suggested to be identified as a biomarker for immune checkpoint inhibitors in pan-cancer [13]. Our study revealed that B4GALNT1 expression was positively correlated with TMB in BRCA (*P* = 1.4*e* − 05), KICH (*P* = 0.013), LIHC (*P* = 0.023), LUAD (*P* = 0.044), LUSC (*P* = 0.027), and THYM (*P* = 6.1*e* − 07), but was negatively associated with TMB in ESCA (*P* = 4.7*e* − 07), GBM (*P* = 0.038), PRAD (*P* = 1.4*e* − 09), STAD (*P* = 1.7*e* − 05), and THCA (*P* = 0.018) (Figure 9(a)).

Mismatch repair (MMR) plays a pivotal role in participating in the correction of base-base mismatch, insertion, and deletion defects during DNA replication. Previous researches have proved a high concordance between MMR deficiency and microsatellite instability (MSI). MSI is a mutation phenotype caused by MMR deficiency and is presented as frequent polymorphism in short repetitive DNA sequences and single nucleotide substitution [14]. Reduction or depletion of MMR might lead to tumorigenesis [15]. Meanwhile, MSI is an important molecular feature of cancers with deficiency in DNA mismatch repairing and has been recognized as a biomarker for the survival and favorable immune checkpoint blockade therapy response in several cancers [16]. Therefore, for the purpose of determining the role of B4GALNT1 in tumorigenesis, we studied the association of B4GALNT1 expression with MSI and MMR.

The relationship of B4GALNT1 expression with MSI in human pan-cancer was presented in Figure 9(b). B4GALNT1 expression had a positive correlation with MSI in BRCA (*P* = 0.011), GBM (*P* = 0.013), HNSC (*P* = 0.048), and UCEC (*P* = 0.00093), while the relationship showed negativity in DLBC (*P* = 0.0064), LAML (*P* = 0.043), STAD (*P* = 0.0029), and TGCT (*P* = 0.018). We also evaluated the association between B4GALNT1 expression and mutation levels of the five MMR genes. The five most important members of the MMR system include MLH1, MSH2, MSH6, PMS2, and EPCAM. The results were presented in Figure 9(c): B4GALNT1 expression was highly linked with MMR genes in multiple types of tumors, including BLCA, BRCA, CESC, COAD, DLBC, ESCA, HNSC, KICH, KIRC, LGG, LIHC, LUAD, LUSC, MESO, OV, PAAD, PCPG, READ, STAD, TGCT, UCEC, UCS, and UVM. The above results indicate that B4GALNT1 possibly regulates tumorigenesis by inducing the defects in DNA mismatch repair.

### 3.6. Methylation

**Figure 10:**
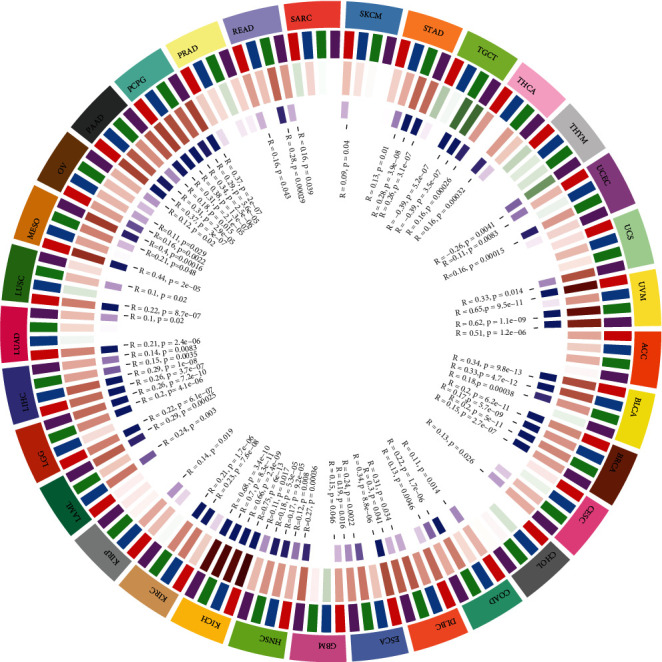
The Spearman's correlation analysis of B4GALNT1 expression with four DNA methyltransferases across cancers. ∗*P* < 0.05, ∗∗*P* < 0.01, and ∗∗∗*P* < 0.001.

Epigenetics is defined as the DNA sequence-independent inheritance of phenotype or gene expression, and DNA methylation is one of the major mechanisms of epigenetic regulation. In various tumors, epigenetic features are often dysregulated, and the accumulation of epigenetic mutation acts as a vital role in tumor progression [17]. Therefore, we studied the association between B4GALNT1 and four DNA methyltransferases (Figure 10). The results showed that B4GALNT1 was related to all four DNA methyltransferases in BLCA, BRCA, COAD, ESCA, HNSC, KICH, LGG, LIHC, MESO, OV, PAAD, PCPG, READ, STAD, and UVM. These results suggested that B4GALNT1 might participate the tumorigenesis possibly by mediating DNA methylation in multiple types of tumors.

### 3.7. Enrichment Analysis of B4GALNT1-Related Partners

**Figure 11:**
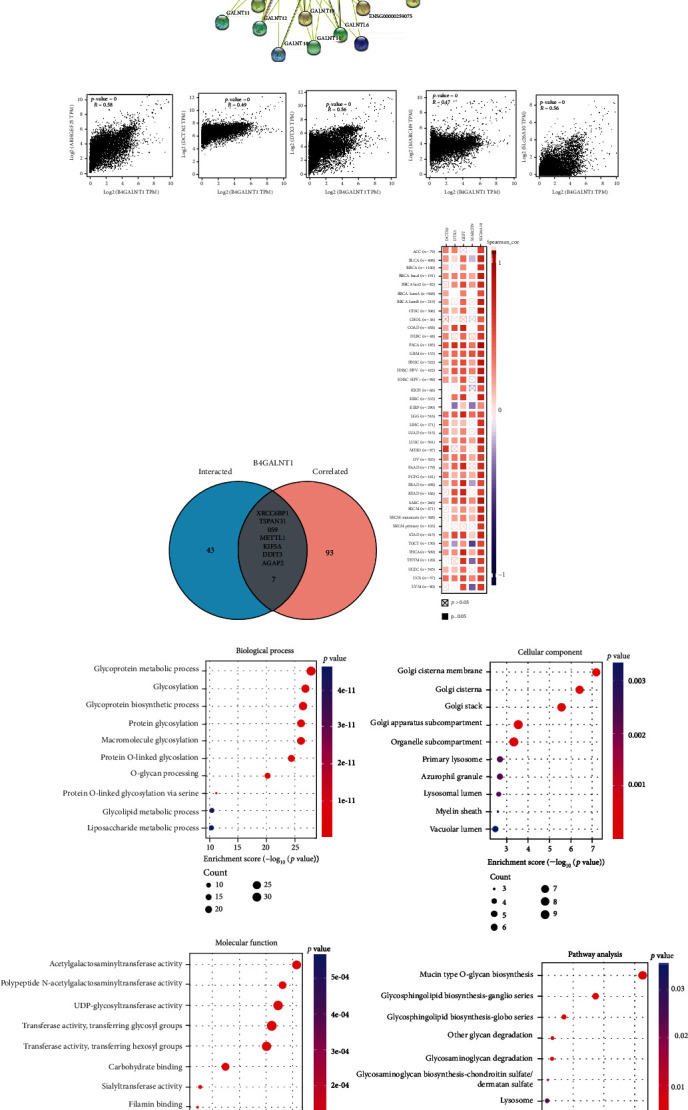
Enrichment analysis of B4GALNT1 expression. (a) An interaction network of 50 B4GALNT1-binding proteins. (b) Correlation between B4GALNT1 expression with ARHGEF25, DCTN2, DTX3, MARCH, and SLC26A10. (c) An intersection of members in (a) and (b). (d) A heat map of correlation between B4GALNT1 expression and the genes in (b). (e) KEGG and GO analyses showed the biological process, cellular component, molecular function, and pathways that were related to B4GALNT1.

To further investigate the underlying molecular mechanism of the B4GALNT1 gene in tumor progression, we attempted to screen out the targeting B4GALNT1-binding proteins and the B4GALNT1 expression-related genes in order to establish a series of pathway enrichment analyses. By applying the STRING tool, we obtained a total of 50 B4GALNT1-binding proteins and presented the interaction network of these proteins (Figure 11(a)). We then used the GEPIA2 tool to integrate all tumor expression data of TCGA and achieved the top 100 genes that correlated with B4GALNT1 expression (Figure 11(b)). The B4GALNT1 expression level was positively correlated with that of ARHGEF25 (or GEFT) (*R* = 0.58), DCTN2 (*R* = 0.49), DTX3 (*R* = 0.56), MARCH9 (*R* = 0.47), and SLC26A10 (*R* = 0.56) genes (all *P* < 0.001). The corresponding heat map data also showed a positive correlation between B4GALNT1 expression and the above five genes in most cancer types (Figure 11(d)). An intersection analysis of the above two groups showed seven common members, namely, XRCC6BP1, TSPAN31, OS9, METTL1, KIF5A, DDIT3, and AGAP2 (Figure 11(c)).

Subsequently, we performed KEGG and GO enrichment analyses in the combined two datasets. The KEGG enrichment analysis data of Figure 11(e) suggested that “glycoprotein metabolic process,” “glycosylation,” “glycoprotein biosynthetic process,” “Golgi cisterna membrane,” “Golgi cisterna,” “Golgi stack,” “acetylgalactosaminyltransferase activity,” “polypeptide N-acetylgalactosaminyltransferase activity,” “UDP-glycosyltransferase,” “Mucin type O-Glycan biosynthesis,” and “glycosphingolipid biosynthesis (ganglio and globo series)” may be involved in the role of B4GALNT1 in tumorigenesis. The GO enrichment analysis data also further indicated that most of these genes are linked to the pathways or cellular biology of the lysosome (Figure 11(e)).

**Figure 12:**
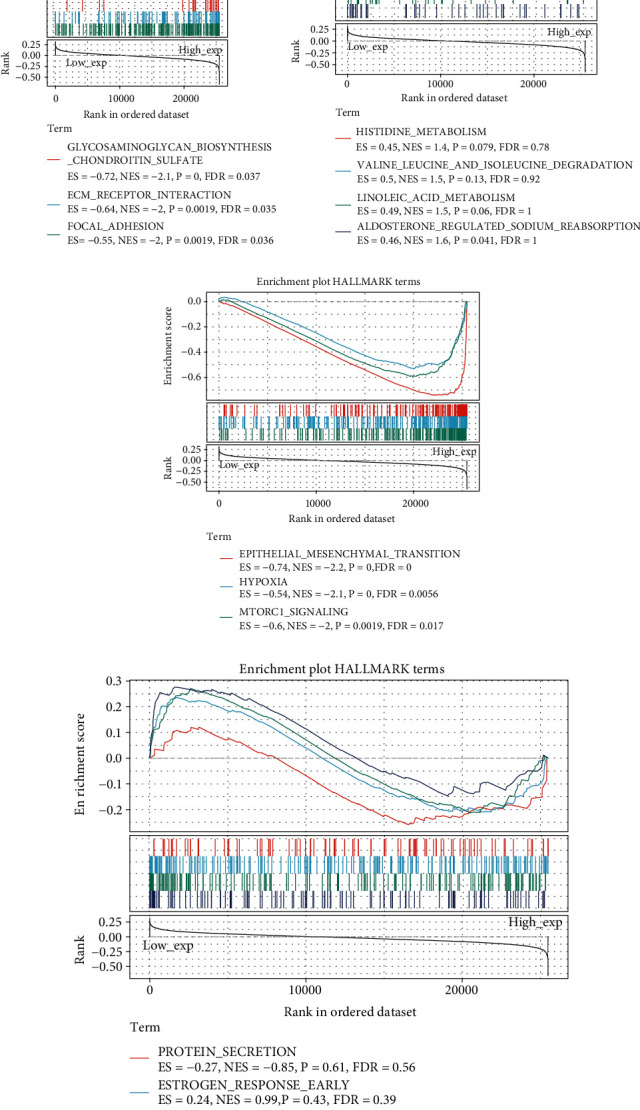
(a–d) KEGG and HALLMARK terms of B4GALNT1 in diverse cancers.

Additionally, we analyzed KEGG and HALLMARK terms of B4GALNT1 in pan-cancer. As shown in Figure 12, B4GALNT1 expression was negatively correlated with Glycosaminoglycan biosynthesis, ECM receptor interaction, and focal adhesion, while its expression was positively correlated with aldosterone-regulated sodium reabsorption. In HALLMARK terms, B4GALNT1 expression was negatively associated with epithelial-mesenchymal transition, hypoxia, and mtorc1 signaling.

### 3.8. Drug Sensitivity Analysis

**Figure 13:**
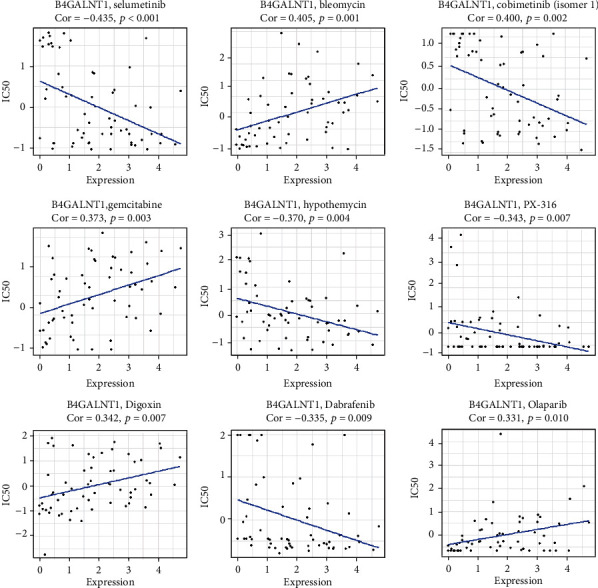
Top 9 correlations between B4GALNT1 and drug sensitivity.

The top 9 correlations between B4GALNT1 and drug sensitivity are shown in Figure 13. The sensitivity of drugs that affect DNA replication and synthesis, such as bleomycin and gemcitabine, was positively correlated with B4GALNT1 expression and paired mRNA expression. These results further indicated the possible clinical usage of B4GALNT1 as a target. Moreover, as for some protein kinase-targeted drugs, such as selumetinib, cobimetinib, and dabrafenib, B4GALNT1 expression showed a negative correlation with the sensitivity.

## 4. Discussion

Beta-1,4-N-Acetyl-Galactosaminyltransferase 1 (B4GALNT1) is a key enzyme that synthesizes GD2/GM2 by transferring an N-acetylgalactosamine (GalNAc) to GD3/GM3 in the ganglioside biosynthesis. Gangliosides are components on the surface of eukaryotic cell membranes, participating in cellular communication, cell adhesion, growth, and differentiation [5].

Previous studies reported that B4GALNT1 was associated with the development of various types of cancers. Its role in tumorigenesis has been investigated: Jiang et al. conducted experiments, revealing that B4GALNT1 induces metastasis and epithelial-mesenchymal-transition (EMT) in LUAD by activating JNK/c-Jun/Slug pathway [18]. Moreover, the enzymatic activity of B4GALNT1 also acts as a major role in tumor metastasis: B4GALNT1 is an enzyme essential for the synthesis of gangliosides such as GD2/GM2, which are proven to be highly expressed in various types of tumor tissues compared with their corresponding normal tissues [5]. Therefore, the function of B4GALNT1 in tumorigenesis is performed through its enzymatic products [18]. Yoshida et al. conducted experiments by comparing GD2/GM2 overexpressing melanoma cells with B4GALNT1 expression and GD2/GM2 negative melanoma cells without B4GALNT1 expression, and their results showed that the GD2/GM2 overexpressing cells with B4GANT1 expression had significantly higher ability of anchorage-independent growth—a critical factor for tumorigenesis and exacerbation of malignancy—than the GD2/GM2 negative group [3]. Their study, along with a study done by Liu et al., also demonstrated that GD2/GM2 can accelerate tumor growth by triggering angiogenesis and without B4GALNT1, such a process cannot be completed [10]. While previous studies mostly focused on the relationship between gangliosides and tumorigenesis, our study revealed the role of the GD2/GM2 synthase—B4GALNT1 in tumorigenesis.

According to our study based on the TCGA datasets, the expressions of B4GALNT1 in tumor tissues were significantly higher than that in their corresponding normal tissues, including ACC, CHOL, HNSC, KIRC, KIRP, LIHC, LUSC, PAAD, SKCM, and TGCT. In previous studies, higher expressions of gangliosides GD2/GM2 have been found in multiple types of tumors including neuroblastoma, breast cancer, lung cancer [5], and cervical cancer [19], which were consistent with our results as the high expression of GD2/GM2 can result from the high expression of B4GALNT1. Furthermore, the expression level of B4GALNT1 had been examined in tumors including breast cancers [7], cervical cancers [14], head and neck squamous cell carcinoma [20], and clear cell renal cell carcinoma [6]; and the expression level was found to be higher than that in normal tissues, which matched the results of our study.

These findings suggested that B4GALNT1 may be used as a marker for diagnosis in specific types of tumors.

In our study, the prognostic landscape in the pan-cancer analysis was visualized using the dataset “Survival Map,” Univariate Cox Analysis, and Kaplan-Meier plotter. Disease-specific interval, disease-specific survival, overall survival, and progression-free survival were four survival analysis standards to evaluate the prognosis of patients. The Univariate Cox analysis and K-M plotter both indicated that B4GALNT1 expression might lead to poorer prognosis in multiple types of tumors, but the tumor types were different when using different survival analysis standards, this might be caused by lack of adequate follow-up time, limited event rates, biased population distribution, or medical intervention. In a genetic alteration analysis, we observed that in all tumor cases, those with altered B4GALNT1 showed better prognosis in overall survival, disease-specific survival, and progression-free survival, but not disease-free survival. These results indicated that B4GALNT1 can be used as a prognostic biomarker for specific types of tumors, but more relevant researches and more accurate data are required.

The tumor microenvironment (TME) consists of intercellular components, nonmalignant cells, vessels, lymphoid organs or lymph nodes, nerves, and metabolites located at the center, margin, or within the vicinity of the tumor lesion. It is an ecosystem that affects every aspect of tumor biology [21]. Fibroblasts and immune cells are important components of TME. Our study, using the database TIMER, EPIC, MCPCOUNTER, XCELL, and TIDE algorithms, revealed that B4GALNT1 expression in BLCA-LumA, COAD, ESCA, HNSC, HNSC-HPV-, KIRC, PAAD, PRAD, READ, STAD, THCA, and THYM is positively correlated with cancer-associated fibroblasts. Meanwhile, after examining 33 types of tumors from the TIMER database, results showed that B4GALNT1 expression was significantly associated with infiltrating immune cells including B cell, CD8 + T cell, CD4 + T cell, macrophage cell, neutrophil cell, and dendritic cell in BRCA, LGG, PAAD, PRAD, and THYM, but whether the correlation is positive or negative differs between tumor types and cell types. Furthermore, we used ImmuneScore and StromalScore to evaluate the population of infiltrating immune cells in TME. The results revealed that B4GALNT1 expression was related to the population of infiltrating immune cells in 21 and 27 types of tumors, respectively. Additionally, the association between B4GALNT1 and 47 common immune checkpoint genes was examined, and the results indicated that B4GALNT1 expression was correlated with more than 25 of the immune checkpoint genes in UVM, COAD, READ, KICH, and PAAD, which gives us ideas to navigate novel methods of immune treatment for the aforementioned tumors.

In previous studies, B4GALNT1 expression-related gangliosides have been observed to play a vital role in tumor immunity: T cells compose a crucial part of tumor-infiltration lymphocytes, and T cell activation is triggered by their surface molecules, known as TCR, into regions on the cell membrane. These regions are called lipid rafts, of which the formation requires gangliosides to participate in [14, 22]. Therefore, although the specific biological mechanism of how B4GALNT1 regulates tumor-infiltration immune cells and how it affects the tumor microenvironment still lacks enough evidence, its role in tumor immunity deserves further investigation. Moreover, our results revealed that B4GALNT1 had great significance with multiple types of common immune checkpoint genes in pan-cancers. Immunotherapy presented a turning point in the antitumor treatment of cancer types in recent years. Common immune checkpoint targets that have been reported for having a promising effect, including Lymphocyte activation gene-3 (LAG-3), T cell immunoglobulin and ITIM domain (TIGIT), and indoleamine 2,3-dioxygenase [23], have presented significance with B4GALNT1 in several cancers. Therefore, further research to discover the potential of B4GALNT1 in cancer treatment is of great necessity.

Tumor mutation burden is defined as the total number of mutations presented in a tumor specimen per megabase. Tumors are considered genomically driven diseases; therefore, gene mutations result in mutated proteins, which are recognized by the immune cells as “non-self” and can induce an antitumor immune response. Microsatellite instability significantly increases the rate of such mutations, as well as deficiency of mismatch repair. Therefore, TMB, MSI, and MMR have been recognized as tumor biomarkers and indications for immunotherapy applications under some circumstances [24]. These results of our study supported this theory and revealed the relationship between B4GALNT1 expression and TMB, MSI, and MMR.

GO enrichment analysis showed that B4GALNT1-related genes mainly function in lysosome activities, while KEGG enrichment analysis suggested that B4GALNT1-related genes are associated with multiple biosynthesis and metabolism processes, including “glycoprotein metabolic process,” “glycosylation,” “glycoprotein biosynthetic process,” “Golgi cisterna membrane,” “Golgi cisterna,” “Golgi stack,” “acetylgalactosaminyltransferase activity,” “polypeptide N-acetylgalactosaminyltransferase activity,” “UDP-glycosyltransferase,” “Mucin type O-Glycan biosynthesis,” and “glycosphingolipid biosynthesis (ganglio and globo series).” In addition, we found out that ARHGEF25 (or GEFT), DCTN2, DTX3, MARCH9, and SLC26A10 were correlated with B4GALNT1 expression in most cancer types.

## 5. Conclusion

The current study revealed that a correlation of B4GALNT1 expression with prognosis exists in multiple types of cancers. In addition, B4GALNT1 expression may play a role in TME and tumor immunity regulation. Further investigation of the biological mechanisms of its different roles in tumorigenesis and clinical application as a biomarker is still required.

## Figures and Tables

**Table 1:** Information of B4GALNT1 in human pan-cancer.

Characteristics	DFI	DSS	OS	PFI	TMB	MSI	TME	ImmuneScore	StromalScore
ACC		N	N	N			N	N	N
BLCA		N	N	N			P	P	P
BRCA					P	P	P		P
CESC									P
CHOL	N	N	N						
COAD		N					P	P	P
DLBC						N			
ESCA					N			N	P
GBM					N	P	N	N	N
HNSC		N	N	N		P		N	P
KICH		N	N	N	P				
KIRC							P		P
KIRP		N	N				P	P	P
LAML						N			
LGG		N	N	N			N	N	N
LIHC			N		P		P	P	P
LUAD		N	N		P		P		P
LUSC					P		N	N	N
MESO		N	N	N					P
OV		N						N	P
PAAD							P	P	P
PCPG							N		N
PRAD					N		P	P	P
READ							P	P	P
SARC							N	N	
SKCM							P	P	P
STAD					N	N	P		P
TGCT						N		P	N
THCA	N				N		P	P	P
THYM		N			P		P		P
UCEC		N	N			P		N	P
UCS								N	
UVM		N	N	N			P	P	P

(N: negative correlated; P: positive correlated).

## Data Availability

Publicly available datasets were used in this study. The data can be found here: GEO database: http://ncbi.nlm.nih.gov/gds; CCLE database: https://sites.broadinstitute.org/ccle; GTEx database: http://gtexport.org/home; TCGA database: http://gdc.cancer.gov/; TIMER web: https://cistrome.shinyapps.io/timer/; TIMER2 web: http://timer.cistrome.org/; GEPIA web-server: http://gepia2.cancer-pku.cn/#analysis; cBioportal web: https://www.cbioportal.org/; STRING website: https://string-db.org/; cellMiner database: http://discover.nci.nih.gov/cellminer/.

## Abbreviations

ACC: Adrenocortical carcinoma
BLCA: Bladder urothelial carcinoma
BRCA: Breast invasive carcinoma
CESC: Cervical squamous cell carcinoma and endocervical adenocarcinoma
CHOL: Cholangiocarcinoma
COAD: Colon adenocarcinoma
DLBC: Lymphoid neoplasm diffuse large B-cell lymphoma
ESCA: Esophageal carcinoma
GBM: Glioblastoma multiforme
HNSC: Head and neck squamous cell carcinoma
KICH: Kidney chromophobe
KIRC: Kidney renal clear cell carcinoma
KIRP: Kidney renal papillary cell carcinoma
LAML: Acute myeloid leukemia
LGG: Brain lower grade glioma
LIHC: Liver hepatocellular carcinoma
LUAD: Lung adenocarcinoma
LUSC: Lung squamous cell carcinoma
MESO: Mesothelioma
OV: Ovarian serous cystadenocarcinoma
PAAD: Pancreatic adenocarcinoma
PCPG: Pheochromocytoma and paraganglioma
PRAD: Prostate adenocarcinoma
READ: Rectum adenocarcinoma
SARC: Sarcoma
SKCM: Skin cutaneous melanoma
STAD: Stomach adenocarcinoma
TGCT: Testicular germ cell tumors
THCA: Thyroid carcinoma
THYM: Thymoma
UCEC: Uterine corpus endometrial carcinoma
UCS: Uterine carcinosarcoma
UVM: Uveal melanoma.

## Supplementary Materials

Supplementary Materials Supplement 1: correlation between immune infiltration level and B4GALNT1 gene expression in diverse cancer types using TCGA database. (a) Using EPIC, MCPCOUNTER, XCELL, and TIDE algorithms to show the relationship between immune cell infiltration levels and B4GALNT1 expression. (b) Scatterplots of the relationship between tumor purity and immune infiltration level and B4GALNT1 expression in tumors with statistically positive correlations in (a). Supplement 2: data of TIMER database showed the relationship between B4GALNT1 expression and multiple tumor-infiltrating lymphocytes in BRCA, LGG, PAAD, PRAD, and THYM.
