# Supplementary material for: Pan-Cancer Analysis of B4GALNT1 as a Potential Prognostic and Immunological Biomarker
Source: J Immunol Res. 2022 Jul 28;2022:4355890. doi: 10.1155/2022/4355890 (PMC9352475; doi:10.1155/2022/4355890)
Supplement: Supplementary Materials — Supplement 1: correlation between immune infiltration level and B4GALNT1 gene expression in diverse cancer types using TCGA database. (a) Using EPIC, MCPCOUNTER, XCELL, and TIDE algorithms to show the relationship between immune cell infiltration levels and B4GALNT1 expression. (b) Scatterplots of the relationship between tumor purity and immune infiltration level and B4GALNT1 expression in tumors with statistically positive correlations in (a). Supplement 2: data of TIMER database showed the relationship between B4GALNT1 expression and multiple tumor-infiltrating lymphocytes in BRCA, LGG, PAAD, PRAD, and THYM. [file 4355890.f1.pdf]

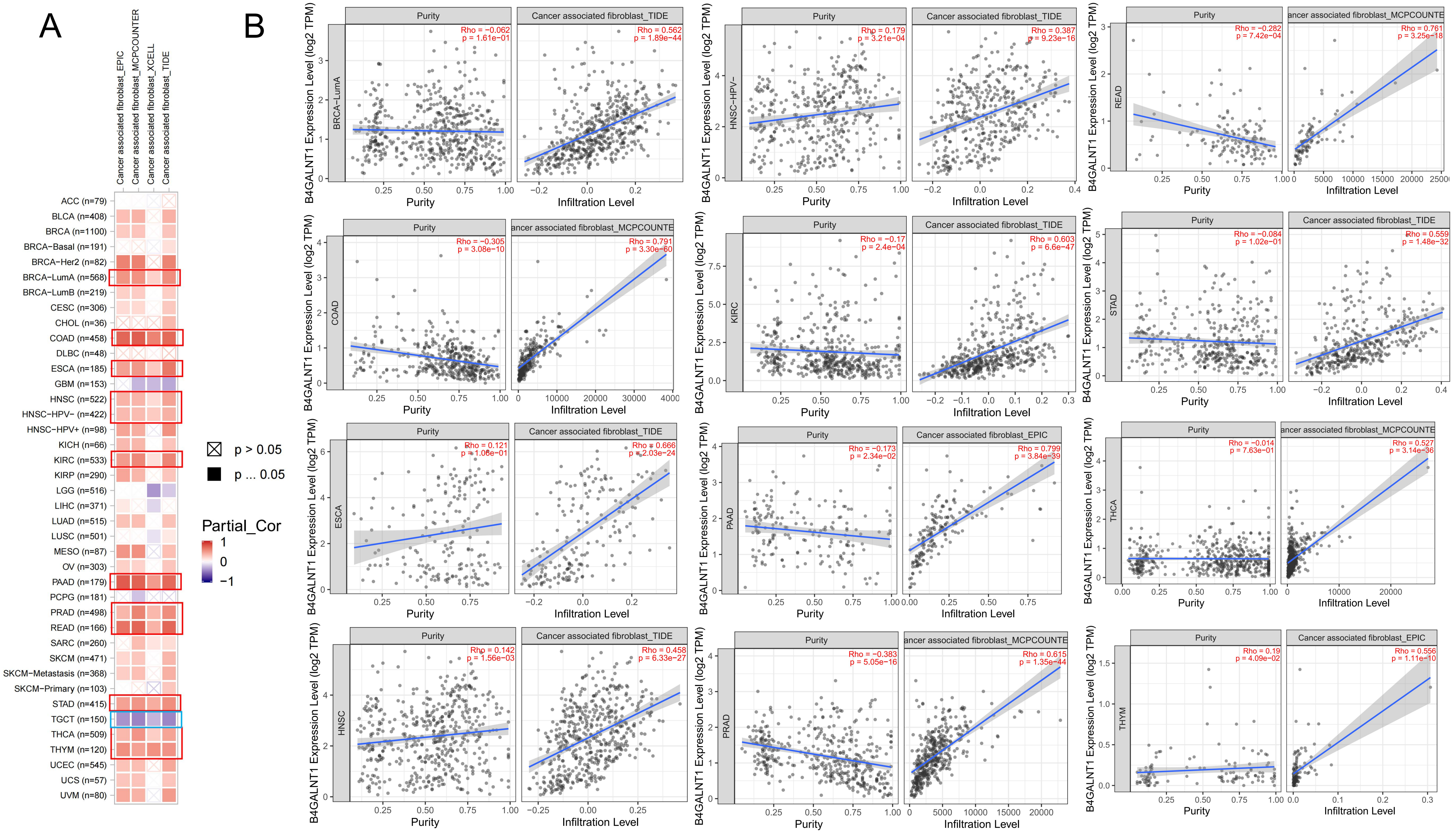

Supplement 1 | Correlation between immune infiltration level and B4GALNT1 gene expression in diverse cancer types using TCGA database. (A) Using EPIC, MCPCOUNTER, XCELL and TIDE algorithms to show the relationship between immune cell infiltration levels and B4GALNT1 expression. (B)Scatterplots of the relationship between tumor purity & immune infiltration level and B4GALNT1 expression in tumors with statistically positive correlations in (A).

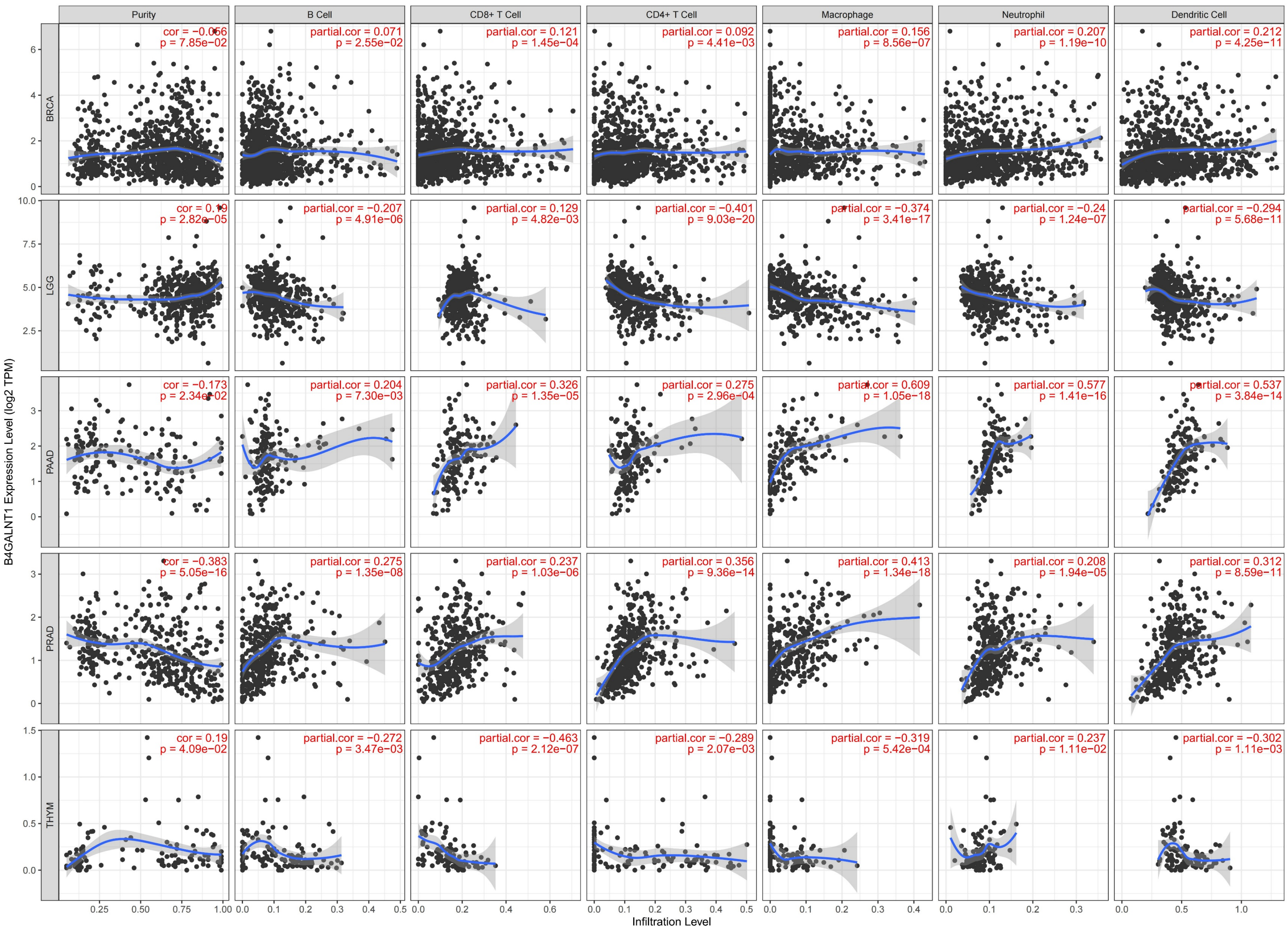

Supplement 2 | Data of TIMER database showed the relationship between B4GALNT1 expression and multiple tumor-infiltrating lymphocytes in BRCA, LGG, PAAD, PRAD and THYM.
